# ADAR1 is a promising risk stratification biomarker of remnant liver recurrence after hepatic metastasectomy for colorectal cancer

**DOI:** 10.1038/s41598-023-29397-z

**Published:** 2023-02-06

**Authors:** Nanako Hata, Kunitoshi Shigeyasu, Yuzo Umeda, Shuya Yano, Sho Takeda, Kazuhiro Yoshida, Tomokazu Fuji, Ryuichi Yoshida, Kazuya Yasui, Hibiki Umeda, Toshiaki Takahashi, Yoshitaka Kondo, Hiroyuki Kishimoto, Yoshiko Mori, Fuminori Teraishi, Hideki Yamamoto, Hiroyuki Michiue, Keiichiro Nakamura, Hiroshi Tazawa, Toshiyoshi Fujiwara

**Affiliations:** 1 Department of Gastroenterological Surgery, Okayama University Graduate School of Medicine, Dentistry, and Pharmaceutical Sciences, 2-5-1 Shikata-Cho, Kita-Ku, Okayama, 700-8558 Japan; 2 Department of Digestive Tract and General Surgery, Saitama Medical Center, Saitama Medical University, Saitama, Japan; 3 Department of Clinical Genomic Medicine, Okayama University Graduate School of Medicine, Dentistry, and Pharmaceutical Sciences, Okayama, Japan; 4 Neutron Therapy Research Center, Okayama University, Okayama, Japan; 5 Department of Obstetrics and Gynecology, Okayama University Graduate School of Medicine, Dentistry, and Pharmaceutical Sciences, Okayama, Japan

**Keywords:** Epigenetics, Colorectal cancer, Metastasis, Tumour biomarkers

## Abstract

Adenosine-to-inosine RNA editing is a process mediated by adenosine deaminases that act on the RNA (ADAR) gene family. It has been discovered recently as an epigenetic modification dysregulated in human cancers. However, the clinical significance of RNA editing in patients with liver metastasis from colorectal cancer (CRC) remains unclear. The current study aimed to systematically and comprehensively investigate the significance of adenosine deaminase acting on RNA 1 (ADAR1) expression status in 83 liver metastatic tissue samples collected from 36 patients with CRC. The ADAR1 expression level was significantly elevated in liver metastatic tissue samples obtained from patients with right-sided, synchronous, or RAS mutant-type CRC. ADAR1-high liver metastasis was significantly correlated with remnant liver recurrence after hepatic metastasectomy. A high ADAR1 expression was a predictive factor of remnant liver recurrence (area under the curve = 0.72). Results showed that the ADAR1 expression level could be a clinically relevant predictive indicator of remnant liver recurrence. Patients with liver metastases who have a high ADAR1 expression requires adjuvant chemotherapy after hepatic metastasectomy.

## Introduction

RNA editing is a mechanism in which the RNA sequence is altered but the DNA sequence is not, thereby resulting in phenotypic changes. The RNA editing enzymes include the ADAR and APOBEC families, which play important roles in embryonic development and immunity^1,2^. Interestingly, RNA editing can promote carcinogenesis^3^. The expression of adenosine deaminase acting on RNA 1 (ADAR1), an RNA editing enzyme, is upregulated in primary colorectal cancer (CRC), and this phenomenon promotes lymph node and distant metastasis. Thus, ADAR1 can be a prognostic marker^4^. In addition, in CRC, cancer-associated fibroblasts receive signals from cancer cells by humoral factors and upregulate RNA editing to promote invasive migration, thereby leading to cancer invasion^5^. Thus, RNA editing can contribute to malignant transformation and can be a potential novel therapeutic target in CRC.

The most important aspect in CRC treatment is distant metastasis control. Recent advancements in chemotherapy have prolonged the life expectancy of patients with CRC who developed distant metastases^6^. Molecular targeted therapy with anti-epidermal growth factor receptor and anti-vascular endothelial growth factor antibodies is associated with a life expectancy of 3 years in unresectable advanced-stage recurrent CRC^7,8^. However, to achieve a longer life expectancy, distant metastatic tumors should be resected without leaving any remnants. In particular, the resection of liver metastatic tumors and the prevention of recurrence are the key to a successful procedure. If liver metastatic tumors can be resected and liver recurrence can be controlled, the life expectancy of patients will be prolonged.

Therefore, the current study aimed to analyze the effects of RNA editing on the development of liver metastasis in patients with CRC and to investigate its therapeutic application. We evaluated the ADAR1 expression of patients with CRC who developed liver metastases via immunostaining. Further, a predictive model for remnant liver recurrence after hepatic metastasectomy was constructed.

## Results

### Remnant liver recurrence after hepatic metastasectomy is associated with a shorter survival

We included a total of 83 resected liver metastases in our study. These liver metastases were resected from 36 patients with CRC liver metastases. Table 1 shows data on the characteristics of patients. The median age of the participants was 68 years. There were 20 male and 16 female patients. In total, 25 patients presented with synchronous liver metastases and 11 with metachronous liver metastases. Further, 11 and 25 patients developed right- and left-sided CRC, respectively, and 16 and 15 patients had RAS mutant- and RAS wild-type CRC, respectively. However, five patients could not be evaluated due to poor DNA quality. The significance of ADAR1 expression in patients with CRC who developed liver metastases was evaluated.

**Table 1:** Characteristics of the patients.

Variables	Number
Age (years)	
< 68	18
≥ 68	18
Sex	
Male	20
Female	16
Type of liver metastasis	
Synchronous	25
Metachronous	11
Number	
< 3	25
≥ 3	11
Sidedness of primary lesion	
Right	11
Left	25
RAS status	
Mutant-type	16
Wild-type	15
Neoadjuvant chemotherapy	
Yes	26
No	10

**Figure 1:**
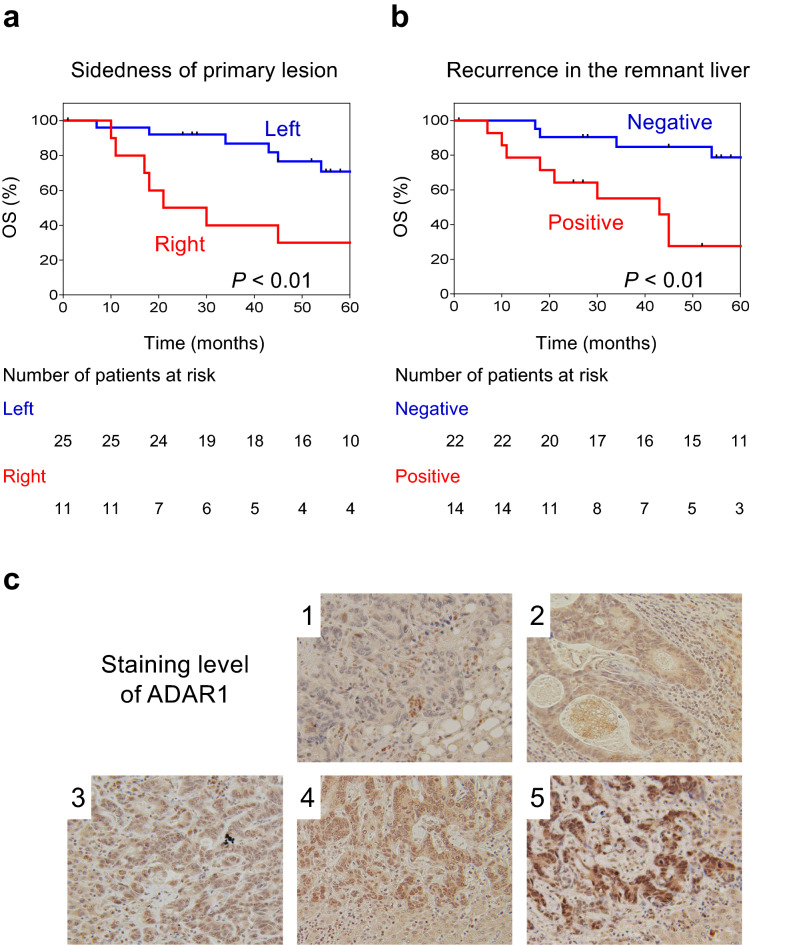
Association between remnant liver recurrence and a shorter survival. (**a**) Patients with liver metastasis from right-sided colorectal cancer had worse overall survival (p < 0.01). (**b**) Patients with remnant liver recurrence after liver metastatic tumor resection had a significantly shorter survival (p < 0.01). (**c**) The level of ADAR1 staining was evaluated using staining scores ranging from 1 to 5.

Patients with right-sided CRC who developed liver metastasis had worse overall survival (p < 0.01; Fig. 1a). Right-sided CRC has a high-malignant potential^9^, and this finding is consistent with our result. In addition, patients with remnant liver recurrence after liver metastatic tumor resection had a significantly short survival (p < 0.01; Fig. 1b). In a multivariate analysis, liver metastasis from right-sided CRC (p = 0.03) and remnant liver recurrence (p = 0.01) were independent predictors of worse prognosis (Table 2).

**Table 2:** Univariate and multivariate analyses of OS.

Variables	Univariate analysis		Multivariate analysis	
	Hazard ratio	p-value	Hazard ratio	p-value
Age ≥ 68 years	1.30	0.62		
Female sex	1.24	0.69		
Synchronous	1.49	0.50		
Number < 3	1.87	0.32		
**Right side**	**4.05**	**0.01**	**3.41**	**0.03**
RAS mutant-type	2.12	0.21		
**Remnant liver recurrence**	**5.45**	**0.01**	**4.90**	**0.01**

Significant values are in bold.

Remnant liver recurrence after hepatic metastasectomy is an indicator of worse prognosis. Further, it is important in identifying patients who are at high risk of remnant liver recurrence and in strengthen treatment application. Therefore, we aimed to identify whether the ADAR1 expression can be a predictive biomarker of remnant liver recurrence after hepatic metastasectomy (Fig. 1c). Predicting the risk of remnant liver recurrence may help in its prevention and the identification of adjuvant chemotherapy indications after liver metastatic tumor resection.

### Analysis of the ADAR1 expression in each metastatic site

**Figure 2:**
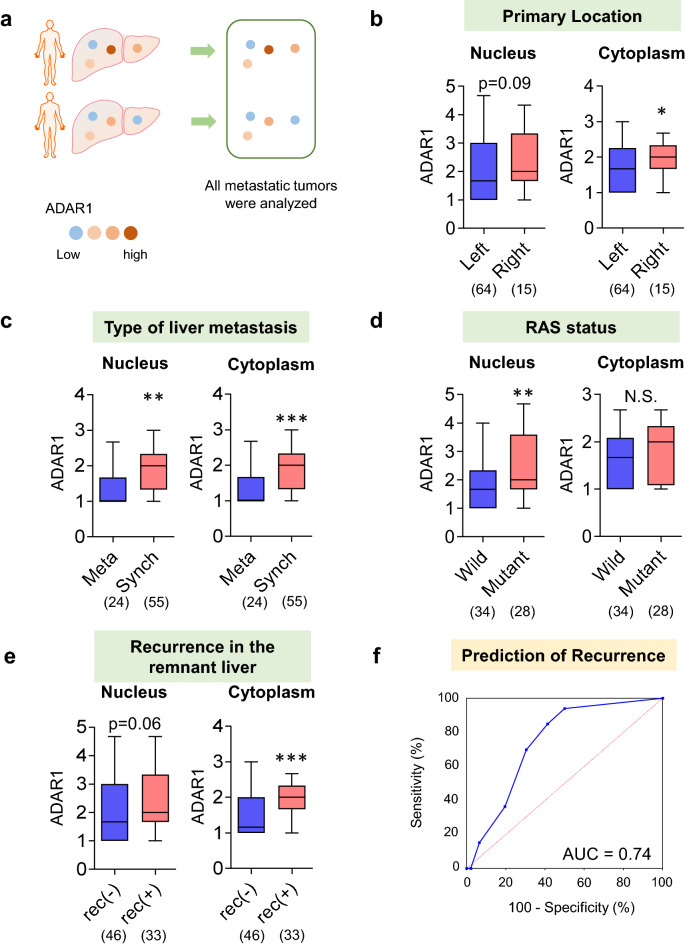
ADAR1 expression in each metastatic site. (**a**) In total, 83 liver metastases were characterized as independent tumor tissues. (**b–e**) The ADAR1 expression was upregulated in patients with liver metastases from right-sided colorectal cancer, concurrent liver metastases, RAS mutant-type cancer, and remnant liver recurrence. (**f**) A high ADAR1 expression was a predictive factor of remnant liver recurrence (area under the curve = 0.72). *p < 0.05, **p < 0.01, ***p < 0.001.

Although most patients had multiple liver metastases, the intensity of ADAR1 immunostaining differed in each tumor. Therefore, we initially characterized 83 liver metastases as independent tumor tissues (Fig. 2a).

The ADAR1 expression in patients with liver metastases was analyzed. Results showed that it was upregulated in patients with liver metastases from right-sided CRC (cytoplasm: p < 0.05; Fig. 2b), concurrent liver metastases (nucleus: p < 0.01, cytoplasm: p < 0.001; Fig. 2c), RAS mutant-type CRC (nucleus: p < 0.01; Fig. 2d), and remnant liver recurrence after hepatic metastasectomy (cytoplasm: p < 0.001; Fig. 2e). Right-sided colon cancer and RAS mutant-type carcinomas are associated with poor prognosis^9^. Interestingly, the current study showed that patients with CRC who had poor prognosis had a high ADAR1 expression. This finding is consistent with the fact that ADAR1 is correlated with increased malignant potential in CRC based on a previous study^4^. Clinically, it is important to predict remnant liver recurrence after hepatic metastasectomy. A high ADAR1 expression was a predictive factor of remnant liver recurrence (area under the curve [AUC] = 0.72; Fig. 2f).

### ADAR1 expression in one representative metastatic site in each patient

**Figure 3:**
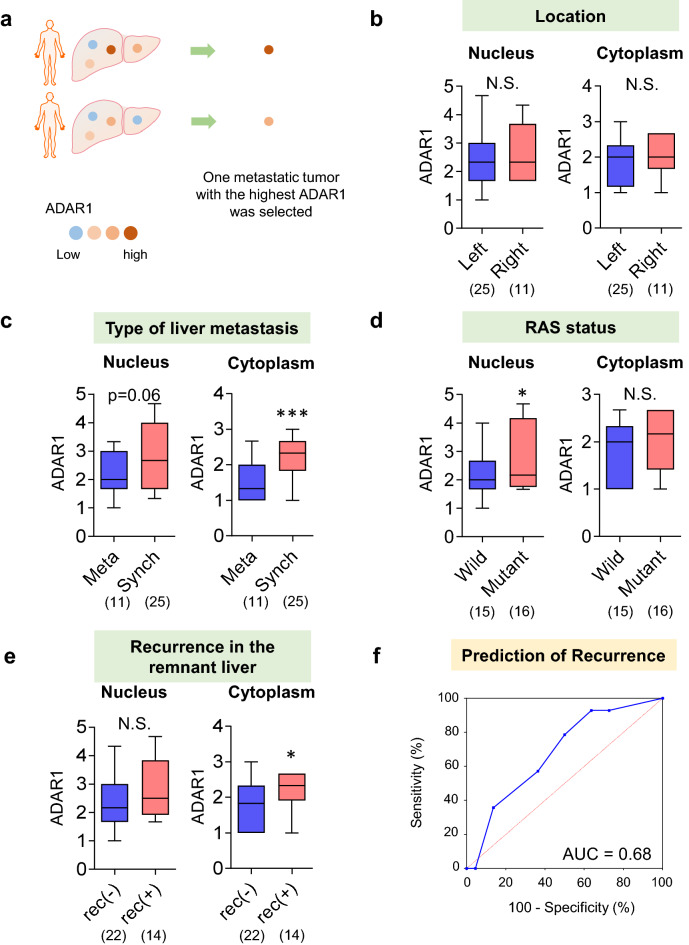
ADAR1 expression in each representative metastatic site in each patient. (**a**) One liver metastasis with the highest ADAR1 staining intensity was selected among liver metastases. (**b–e**) The ADAR1 expression was upregulated in patients with liver metastases from concurrent liver metastases, RAS mutant-type cancer, and remnant liver recurrence. (**f**) A high ADAR1 expression was a predictive factor of remnant liver recurrence (area under the curve = 0.68). *p < 0.05, ***p < 0.001.

If a patient has several liver metastatic tumors, these lesions might have different ADAR1 staining intensities. Averaging the ADAR1 staining intensities could diminish the characteristics of liver metastatic tumors. Therefore, in each patient, we selected one liver metastatic site with the highest ADAR1 staining intensity (Fig. 3a). Then, the association between ADAR1 staining intensity and clinicopathological features was examined.

**Figure 4:**
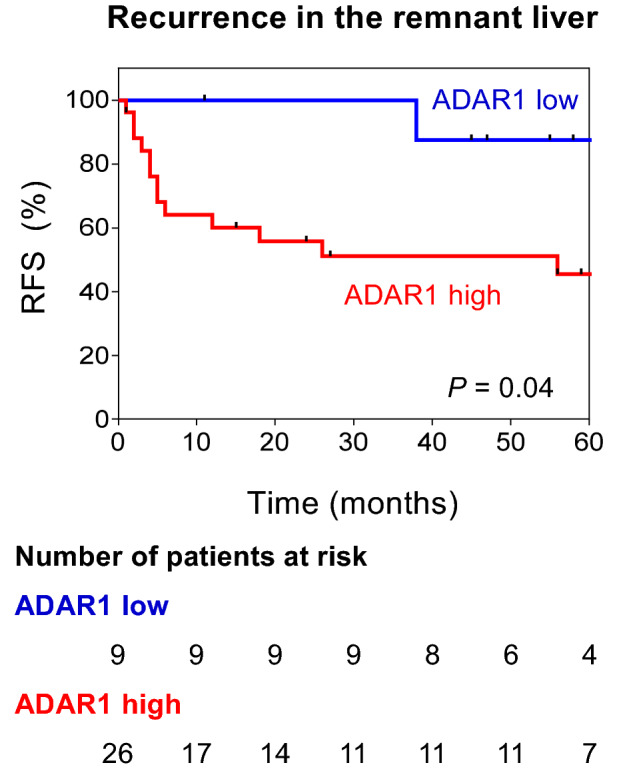
Association between ADAR1 expression and remnant liver recurrence. Patients with liver metastases who have high ADAR1 levels had earlier remnant liver recurrence.

The results were similar to those obtained by analyzing each independent liver metastatic tumor. The ADAR1 expression was upregulated in patients with liver metastases related to concurrent liver metastases (cytoplasm: p < 0.001), RAS mutant-type CRC (nucleus: p < 0.05), and remnant liver recurrence after hepatic metastasectomy (cytoplasm: p < 0.05; Fig. 3b–e). A high ADAR1 expression was a predictive factor of remnant liver recurrence (AUC = 0.68; Fig. 3f). Using the log-rank test, patients with liver metastases who have high ADAR1 levels had earlier remnant liver recurrence after hepatic metastasectomy (p = 0.04; Fig. 4). The multivariate analysis was also performed using the Cox hazard model. Results showed that high ADAR1 levels remained an independent high risk factor for remnant liver recurrence in patients with liver metastases (p = 0.05, Table 3).

**Table 3:** Univariate and multivariate analyses of remnant liver recurrence.

Variables	Univariate analysis		Multivariate analysis	
	Hazard ratio	p-value	Hazard ratio	p-value
**Right side CRC**	**2.93**	**0.06**	2.20	0.17
RAS mutant-type	1.55	0.42		
Synchronous	2.23	0.19		
Number ≥ 3	1.16	0.80		
Size ≥ 30 mm	1.46	0.54		
Margin positive	1.77 × e−9	0.12		
CEA ≥ 10 ng/ml	1.17	0.78		
CA19-9 ≥ 50 U/ml	1.82	0.39		
**High ADAR1 expression**	**6.38**	**0.02**	**5.19**	**0.05**

Significant values are in bold.

Based on these results, we hypothesized the following clinical applications: If patients with liver metastases from CRC undergo surgery, ADAR1 immunostaining should be performed, and the expression intensity must be assessed. Patients whose highest ADAR1 immunostaining intensity exceeds the cutoff value were at high risk of remnant liver recurrence after hepatic metastasectomy.

### Immunostaining results of ADAR1 in the primary lesion cannot be a predictive factor of remnant liver recurrence

**Figure 5:**
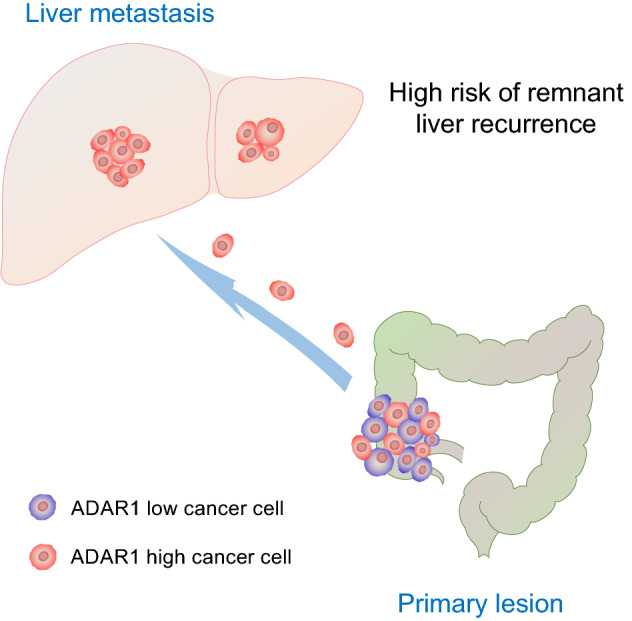
Highly malignant colorectal cancer cells with a high ADAR1 expression cause liver metastases. High-malignant cancer cells with a high ADAR1 expression are more likely to cause liver metastases, and even a small number of cells can easily metastasize, thereby resulting in remnant liver recurrence over time.

We evaluated the expression of ADAR1 in the primary tumor (Supplementary Fig. 1a). However, no correlation with clinicopathological features was observed (Supplementary Fig. 1b–e). Moreover, the predictive ability of ADAR1 expression in the primary tumor was poor (AUC = 0.59; Supplementary Fig. 1f). This finding could be attributed to primary tumor heterogeneity. Thus, primary tumors contain a mixture of cells with high and low ADAR1 expressions. Due to heterogeneity, this was not evident in the analysis of the primary tumor. We hypothesized that highly malignant cancer cells with a high ADAR1 expression are more likely to cause liver metastases, and even a small number of these cells can easily metastasize, thereby resulting in remnant liver recurrence after hepatic metastasectomy (Fig. 5).

## Discussion

Patients with CRC presented with a series of genetic and epigenetic alterations in colon tissues^10^. RNA editing has emerged as an important epigenetic modification involved in the evolution of different types of cancers and disease progression. Adenosine-to-inosine RNA editing, which is associated with oncogenes and tumor suppressor genes, can alter tumor characteristics to promote a more aggressive phenotype^11^. AZIN1, which is aberrant in different types of cancers, is a major target of ADAR1. Further, it is significantly edited in different types of cancers, including hepatocellular carcinoma, esophageal cancer, and CRC^4,11,12^. Emerging evidence has shown that edited AZIN1 is highly oncogenic, and it inhibits ornithine decarboxylase degradation and induces polyamine accumulation and invasion, migration, and stemness^12^.

On the other hand, Liver metastasis in CRC requires special attention. Patients with CRC who developed liver metastases have poor prognosis. The median survival of patients with hepatic metastasis from CRC who did not receive treatment is 5–20 months^13^. The prognosis is extremely poor if liver metastatic tumors become unresectable, particularly in right-sided CRC, which has a 5-year OS rate of 4.3%^14^. Therefore, CRC treatment aims to control liver metastases.

High-grade malignant liver metastases are more likely to be correlated with remnant liver recurrence and extrahepatic lymph node metastases. Such extremely malignant liver metastases are challenging to control by surgery alone, and they require preoperative or postoperative chemotherapy^15^. However, grading of liver metastases from CRC is still technically challenging.

The current study had an extremely important finding. ADAR1 was highly expressed in patients with liver metastases from CRC, which resulted in remnant liver recurrence after hepatic metastasectomy. Patients with CRC who developed ADAR1-expressing liver metastases had an earlier and higher rate of remnant liver recurrence. Thus, ADAR1 immunostaining at the time of liver metastasis resection may identify patients at high risk for remnant liver recurrence after hepatic metastasectomy. The results of the current study will be useful in the evaluation of adjuvant chemotherapy indications after liver metastatic tumor resection.

To date, the need for adjuvant chemotherapy after liver metastatic tumor resection in CRC remains controversial^6^. Most recently, the JCOG 0603 trial was conducted, and the results were as follows: from March 2007 to January 2019, 300 patients were randomly assigned to undergo either liver resection-alone or liver resection, followed by adjuvant chemotherapy^16^. In the combined phase II and phase III study, 149 patients were included in the surgery alone group and 151 in the chemotherapy group. The 5-year disease-free survival rates were 38.7% in the liver resection-alone group and 49.8% in the adjuvant chemotherapy group. That is, the adjuvant chemotherapy group had a better disease-free survival than the liver resection-alone group. By contrast, the adjuvant chemotherapy group had a lower 5-year overall survival rate than the liver resection-alone group (71.2% vs. 83.1%). This controversial result may be attributed to the fact that there are no adequate eligibility criteria for adjuvant therapy. The ADAR1 expression can accurately predict remnant liver recurrence after hepatic metastasectomy in patients with liver metastases. Thus, more intensive adjuvant chemotherapy may be effective in patients with liver metastases with a high ADAR1 expression. The ADAR1 expression may affect the choice of adjuvant therapy protocol.

This retrospective study had several limitations. That is, it was performed at a single center. We are currently planning to perform a clinical trial to reduce remnant liver recurrence with adjuvant chemotherapy in patients with liver metastases who have a high ADAR1 expression, and we hope to report the results in the near future.

In conclusion, a high ADAR1 expression is associated with a greater risk of remnant liver recurrence after hepatic metastasectomy in patients with liver metastases from CRC. Therefore, it may be a good indicator of multimodality treatment, including chemotherapy, in patients with liver metastases from CRC who have high ADAR1 expression levels.

## Methods

### Patients and sample collection

This study examined 83 cases of liver metastases resected from 36 patients at Okayama University. Patients who did or did not receive neoadjuvant chemotherapy were both included. CRC diagnosis was confirmed in all patients based on clinicopathological findings. The Tumor Node Metastasis staging system of the American Joint Committee on Cancer was used for pathological staging. The current research was approved by the Ethics Committee of Okayama University Graduate School of Medicine, Dentistry, and Pharmaceutical Sciences and Okayama University Hospital (1903-037). A written informed consent was obtained from each patient. All methods were performed in accordance with the relevant guidelines and regulations.

### Immunohistochemical analysis

Paraffin-embedded sections were deparaffinized using xylene and ethanol, and endogenous peroxidase activity was eliminated with H_2_O_2_, as previously described^5^. After antigen retrieval by autoclaving the tissues at 121 °C for 15 min, the slides were incubated overnight with an anti-ADAR1 antibody at a 1:100 dilution (Abcam, Cambridge, MA, the USA). Color development was achieved using the EnVision + Dual Link Kit (DAKO, Carpinteria, CA, the USA), and the slides were counterstained with hematoxylin. Negative controls were run in parallel. The level of ADAR1 staining was evaluated using the staining score ranging from 1 to 5^4^ and measured three times by three independent investigators who were blinded to the nature of the specimens and antibodies used.

### Statistical analysis

Data were expressed as mean ± standard deviation. The JMP software (version 10.0, SAS Institute Inc., Cary, NC, the USA) was used to perform statistical analyses. Between-group differences were assessed using the Wilcoxon’s rank-sum test, χ^2^ test, and Steel test, as appropriate. The correlations between two groups were evaluated via Spearman’s rank correlation analysis. For time-to-event analyses, survival estimates were calculated using the Kaplan–Meier method, and groups were compared with the log-rank test. Two-sided p-values of < 0.05 were considered statistically significant.

### Ethics approval and consent to participate

A written informed consent was obtained from each patient, and the current study was approved by the Ethics Committee of Okayama University Graduate School of Medicine, Dentistry, and Pharmaceutical Sciences and Okayama University Hospital (1903-037).

## Supplementary Information

Supplementary Figure 1.

## Data Availability

All data generated or analyzed during this study are included in the published article.
