# Supplementary material for: ADAR1 is a promising risk stratification biomarker of remnant liver recurrence after hepatic metastasectomy for colorectal cancer
Source: Sci Rep. 2023 Feb 6;13:2078. doi: 10.1038/s41598-023-29397-z (PMC9902515; doi:10.1038/s41598-023-29397-z)
Supplement: Supplementary file 1 — Supplementary Figure 1. [file 41598_2023_29397_MOESM1_ESM.docx]

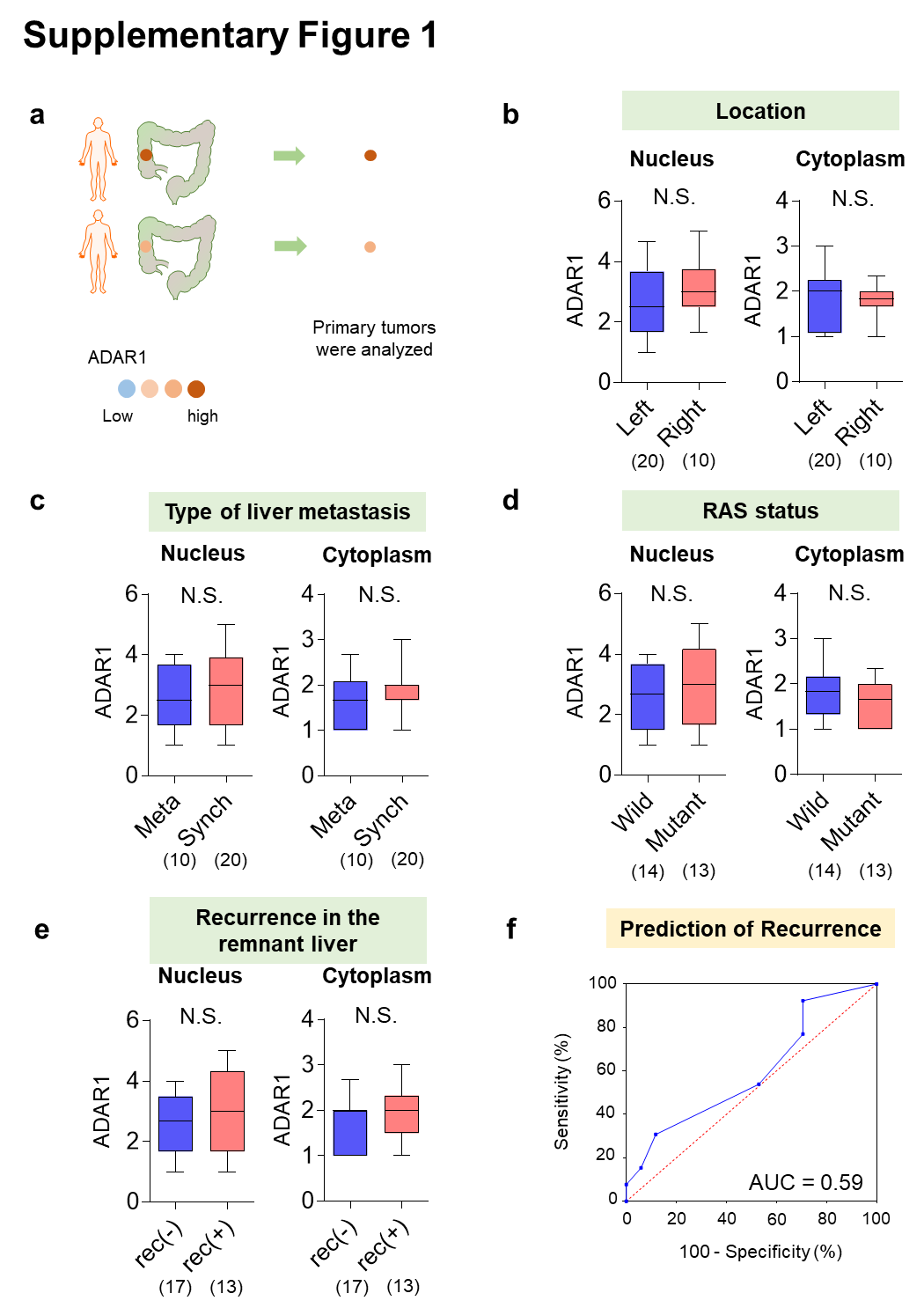


**(a)** The ADAR1 expression in the primary tumor was analyzed. **(b–e)** No correlation was observed between ADAR1 expression in the primary tumor and clinicopathological status. **(f)** The predictive ability of ADAR1 expression in the primary tumor for remnant liver recurrence was poor (area under the curve = 0.59).
